# Deep-Infiltrating Endometriosis Causing Acute Mechanical Intestinal Obstruction Without Intestinal Invasion: A Case Report with Diagnostic and Surgical Insights

**DOI:** 10.3390/jcm15041664

**Published:** 2026-02-23

**Authors:** Jung Hyun Park, Jeonghyeon Shin, Mee-Ran Kim

**Affiliations:** Division of Reproductive Endocrinology, Department of Obstetrics and Gynecology, College of Medicine, The Catholic University of Korea, Seoul 06591, Republic of Korea

**Keywords:** endometriosis, endometrioma, deep-infiltrating endometriosis, intestinal obstruction, ileus, laparoscopy

## Abstract

Background: Endometriosis is a chronic, estrogen-dependent disorder that may extend beyond the pelvis to involve the gastrointestinal tract, most commonly the rectosigmoid and, less frequently, the small bowel. Although often asymptomatic, such lesions may rarely manifest as acute bowel obstruction. Case: We report a 42-year-old woman who presented with small bowel ileus caused by deep-infiltrating endometriosis (DIE). Imaging revealed a right ovarian endometrioma with severe adhesions resulting in a distal ileal transition point. After partial decompression with conservative treatment, laparoscopic adhesiolysis with right salpingo-oophorectomy and left salpingectomy was undertaken. Intraoperative findings revealed dense adnexal–ileal adhesions without transmural involvement. Postoperative hormonal suppression was instituted. Conclusions: This rare case demonstrates small bowel obstruction caused by DIE adhesions without intestinal invasion. Preoperative imaging facilitated a minimally invasive approach, while combined surgical and hormonal therapy was associated with reduced recurrence risk. These findings expand the recognized spectrum of endometriosis-related intestinal complications and support tailored management strategies.

## 1. Background

Endometriosis is a chronic, estrogen-dependent condition characterized by ectopic endometrial tissue, with Sampson’s theory of retrograde menstruation being the most widely accepted explanation of its pathogenesis [1]. The prevalence of endometriosis ranges from 2 to 10% in the general population and up to 35% among women of reproductive age, most commonly between 25 and 45 years [2]. While the genital tract is predominantly affected, extragenital involvement occurs in the gastrointestinal (GI) tract (3–37%) and urinary tract (~10%), with rare cases reported in extra-abdominal sites such as the lungs, perineum, skin, and central nervous system [3]. Intestinal endometriosis (IE) is usually asymptomatic or presents with nonspecific GI symptoms, most commonly involving the rectosigmoid (50–90%), followed by the small bowel (2–16%), appendix (3–18%), and cecum (2–5%) [4].

Intestinal obstruction is an uncommon but serious complication of IE, occurring in fewer than 1% of cases, with small bowel obstruction accounting for only 0.7% of all surgical interventions [5]. Obstruction typically results from extrinsic compression, adhesions, or stricture formation rather than intraluminal masses, most often involving distal ileal implants that cause angulation or kinking of bowel loops. Intestinal perforation represents an even rarer but life-threatening manifestation, with approximately 20 cases reported, predominantly during pregnancy or the postpartum period [6]. Decidualization of endometriotic implants under high progesterone levels, together with mechanical traction from the enlarging uterus, is considered the major mechanism underlying these pregnancy-associated perforations [7].

Although surgical intervention is not considered the primary treatment for endometriosis, emergency surgery becomes indispensable when intestinal complications occur. We present a rare case of acute small bowel obstruction secondary to deep-infiltrating endometriosis (DIE), managed with emergency surgical intervention, with definitive diagnosis established through histopathological examination of the resected adhesion barrier between the adnexa and bowel.

## 2. Case Presentation

A 42-year-old gravida 2 woman was admitted to the emergency department with two weeks of diffuse abdominal pain and vomiting. The last normal defecation was confirmed on the morning of the same day, and flatus passage was preserved. The patient had no previously diagnosed comorbidities or surgical history, reported no history of medication use, including hormonal treatments, and both previous deliveries were spontaneous vaginal births. The patient reported a regular 28-day menstrual cycle, with the first day of her last menstrual period occurring 20 days before presentation, consistent with the luteal phase. Apart from the 2-week history of abdominal pain at presentation, she denied any baseline pelvic pain symptoms, including dysmenorrhea, menorrhagia, or dyspareunia. On presentation, she was hemodynamically stable with a body temperature of 37.2 °C. Her body mass index was 17.8 kg/m^2^ (height, 171 cm; weight, 52 kg).

Physical examination revealed diffuse abdominal distension with hypoactive bowel sounds and localized tenderness in the right lower quadrant. Rectal examination was unremarkable. Laboratory investigations were within normal limits, with a hemoglobin level of 14.7 g/dL, although the cancer antigen 125 (CA-125) was elevated at 67.4 U/mL.

**Figure 1:**
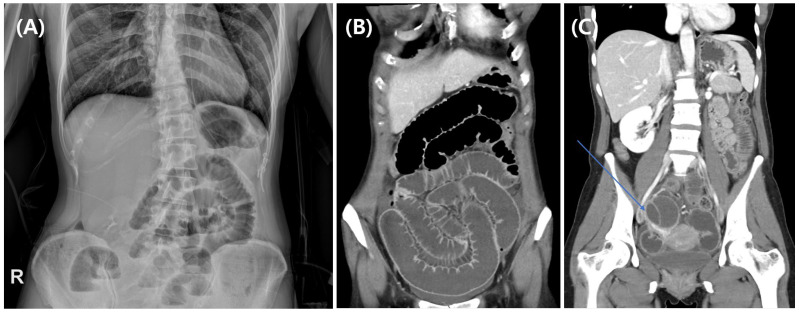
(**A**) Upright abdominal radiograph demonstrating dilated small bowel loops with multiple air–fluid levels. The “R” marker indicates the patient’s right side. (**B**) Coronal abdominal CT image showing dilated small bowel loops. (**C**) Axial abdominal CT image revealing a 5.5 × 5.0 cm right-sided ovarian cyst suggestive of endometrioma (blue arrow), with a transition point at the distal ileum in the pelvic cavity due to adhesion with the right adnexal cystic mass. Abbreviations: CT; computed tomography.

**Figure 2:**
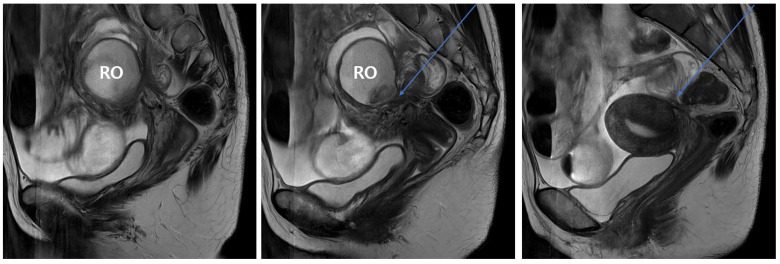
Serial sagittal pelvic MRI images demonstrating a 5.1 cm multiloculated cystic lesion in the right ovary (RO) with T1 hyperintensity and T2 shading, consistent with an endometrioma. Severe adhesions are observed among the right ovary, rectosigmoid colon, and posterior uterine wall. The right endometrioma shows dense adhesion to the distal ileum with a whirling appearance and a transition point (blue arrows), resulting in small bowel obstruction. Findings are consistent with deep-infiltrating endometriosis. Abbreviations: MRI, magnetic resonance imaging, RO, right ovary.

An upright abdominal radiograph demonstrated dilated small bowel loops with multiple air–fluid levels (Figure 1A). Intravenous contrast-enhanced abdominal computed tomography (CT) confirmed small bowel obstruction with ascites, along with a 5.5 × 5.0 cm right ovarian endometrioma (Figure 1B,C). Given these findings, pelvic magnetic resonance imaging (MRI) was performed to exclude malignancy. MRI revealed a right ovarian endometrial cyst accompanied by severe adhesions between the right ovary, rectosigmoid colon, and uterus due to DIE, as well as an adhesion point at the distal ileum resulting in a small bowel ileus with a transition point (Figure 2).

The patient initially underwent conservative management with nasogastric decompression, antibiotics, and fluid resuscitation. During this period, she remained hemodynamically stable without fever, and serial laboratory evaluations demonstrated normal white blood cell counts and C-reactive protein levels. Four days later, after partial improvement with decompression, a diagnostic laparoscopy was planned to address the underlying etiology of the ileus.

**Figure 3:**
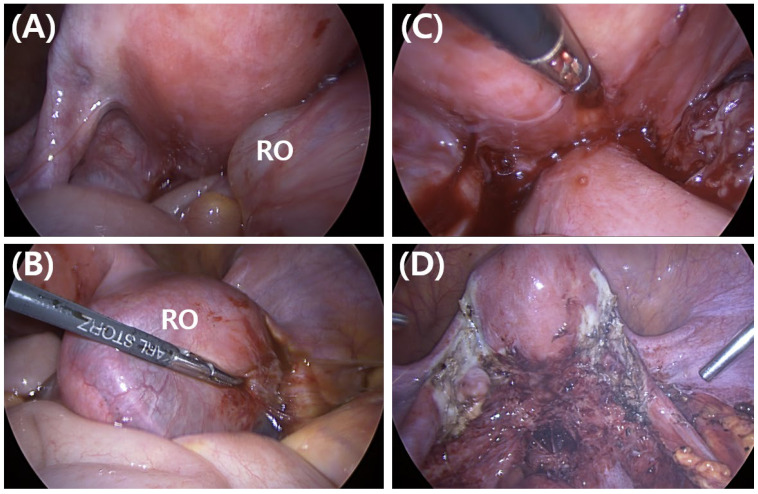
Intraoperative laparoscopic findings. (**A**,**B**) Dense adhesions involving the right ovary (RO), uterus, appendix, and ileum. (**C**) The distal ileum adheres to the posterior uterine wall, rectosigmoid colon, and uterosacral ligament. (**D**) Postoperative view after right salpingo-oophorectomy, left salpingectomy, adhesiolysis, and fulguration of visible pelvic endometriotic lesions. Abbreviations: RO, right ovary.

A four-port laparoscopy was performed (10 mm umbilical, 5 mm right and left lower quadrants, and 5 mm suprapubic), during which approximately 150 mL of ascites was identified and subsequently confirmed negative on cytological examination. At initial laparoscopic inspection, dense adhesions were noted among the right adnexa, uterus, appendix, and ileum (Figure 3A,B). The distal ileum was adherent to the posterior uterine wall, rectosigmoid colon, and uterosacral ligament, while the left adnexa was adherent to both the uterus and rectum (Figure 3C).

Extensive adhesiolysis was performed, followed by meticulous inspection of the entire small bowel. No gross evidence of transmural endometriotic invasion into the intestinal wall was identified, and bowel resection was therefore not indicated. Similarly, no gross evidence of appendicitis was observed; appendectomy was deemed unnecessary. Given that the patient firmly denied plans for future childbearing and intraoperative findings demonstrated endometriotic spots on both adnexal surfaces, including the fallopian tubes, a right salpingo-oophorectomy and left salpingectomy were performed to reduce the risk of recurrence, along with electrocautery fulguration of visible pelvic endometriotic lesions (Figure 3D). Based on intraoperative findings, the cumulative score according to the revised American Society for Reproductive Medicine staging system was 122 points, consistent with stage IV endometriosis. Before completing the procedure, intraoperative cystoscopy with intravenous indigo carmine administration confirmed the absence of ureteral injury.

Histopathological examination confirmed endometriosis within the adhesion bands, both fallopian tubes, and the right adnexa. The patient had an uneventful recovery, with drain removal and dietary advancement achieved by postoperative day 6. She was discharged on postoperative hormonal therapy, consisting of a gonadotropin-releasing hormone agonist (GnRHa) injection followed by Dienogest administration for long-term suppression of recurrence. At the 3-month follow-up, clinical evaluation and imaging studies showed no signs of recurrence, with normalization of serum CA-125 levels.

## 3. Discussion

We present a case of acute mechanical intestinal obstruction secondary to adhesions from DIE, which was effectively managed through conservative decompression and subsequent laparoscopic adhesiolysis. Our case exceptionally illustrates ileus induced solely by DIE without intestinal invasion, contrasting with the rare (<1%) endometriosis-associated obstructions that typically involve IE [8].

By documenting this rare presentation, our report addresses a gap in the limited number of reported cases and contributes to a more comprehensive recognition of the diverse clinical manifestations of DIE. To begin with, all previously reported cases, retrospective analyses, and reviews of endometriosis-induced intestinal obstruction involved concomitant IE, and surgical management invariably required bowel resection [1,3,8,9,10]. In contrast, our patient represents a novel case of acute intestinal obstruction necessitating emergency surgery in the absence of IE. Remarkably, neither bowel resection nor appendectomy was required, as no transmural invasion or appendicitis was identified. The absence of prior abdominal surgery raised concern regarding whether ileus could be solely attributed to a right ovarian endometrioma and DIE; however, pelvic MRI demonstrated a bowel transition point caused by adhesion to the adnexal mass, thereby justifying surgical intervention. Furthermore, in the review by Mușat et al., 81 of 107 patients with endometriosis-related intestinal obstruction were diagnosed with endometriosis for the first time at the occlusive event [8]. Likewise, our patient, despite regular biannual transvaginal ultrasonography, had no prior diagnosis of endometrioma and was first identified with DIE during the obstruction episode. A limitation of this case is the short follow-up period of three months, which is insufficient to assess long-term recurrence in endometriosis. Longer-term surveillance is required to evaluate the durability of treatment.

Our therapeutic approach emphasized two critical strategies aimed at minimizing postoperative recurrence. First, as retrograde menstrual flow through the fallopian tubes is regarded as a key mechanism for the peritoneal dissemination of endometrial tissue [11], bilateral salpingectomy was undertaken along with extensive adhesiolysis and right oophorectomy at diagnostic laparoscopy. In carefully selected patients who have definitively completed childbearing, bilateral salpingectomy may be considered as part of an individualized surgical strategy, particularly when intraoperative findings demonstrate extensive adnexal involvement. Furthermore, unilateral oophorectomy may be justified under the surgeon’s prudent judgment in cases of severe ovarian disease or high risk of recurrence, given that preservation of the contralateral ovary generally maintains endocrine function and does not typically induce premature menopause [12].

Second, postoperative medical hormonal therapy was instituted to further reduce the risk of recurrence. This strategy is supported by prior evidence reporting re-operation rates as high as 27–58% following endometriosis surgery with ovarian preservation [13]. After an initial short course of GnRHa injection for rapid postoperative suppression, we transitioned to Dienogest as maintenance therapy to sustain long-term disease control with a more favorable tolerability profile than continued hypoestrogenic GnRHa exposure [14]. In line with guidance supporting long-term postoperative hormonal therapy in women not seeking immediate conception and evidence that Dienogest 2 mg can be used effectively and safely for up to 60 months [15], we planned to continue medication until the expected age of natural menopause unless contraindications or intolerance occur [16]. Collectively, the surgical and medical approach in this case highlights a comprehensive strategy that not only addresses the acute complication but also targets the underlying mechanisms of recurrence, thereby reinforcing its clinical significance.

## 4. Conclusions

In conclusion, we report a rare case of acute intestinal obstruction caused solely by DIE without intestinal involvement. In contrast to prior reports, our patient required neither bowel resection nor appendectomy, highlighting the value of preoperative imaging in guiding tailored surgical decision-making. Initial conservative decompression permitted a minimally invasive laparoscopic approach, which is recommended whenever feasible to reduce morbidity. Finally, the combined strategy of targeted surgery and postoperative hormonal therapy underscores the importance of reducing endometriosis recurrence, thereby broadening the recognized clinical spectrum of endometriosis-related intestinal complications.

## Data Availability

The original contributions presented in the study are included in the article. Further inquiries can be directed at the corresponding author.

## Abbreviations

The following abbreviations are used in this manuscript:

GI	Gastrointestinal
IE	Intestinal endometriosis
DIE	Deep-infiltrating endometriosis
CA-125	Cancer antigen 125
CT	Computed tomography
MRI	Magnetic resonance imaging
GnRHa	Gonadotropin-releasing hormone agonist
